# Advanced Bioluminescence Reporter with Engineered *Gaussia* Luciferase via Sequence-Guided Mutagenesis

**DOI:** 10.3390/bios14110528

**Published:** 2024-11-01

**Authors:** Vinayakumar Gedi, Eun Hye Kim, Bohyun Oh, Young-Pil Kim

**Affiliations:** 1 Department of Life Science, Hanyang University, Seoul 04763, Republic of Korea; vinayreddygedi@outlook.com (V.G.); yomi135@naver.com (E.H.K.); bohyun5180@naver.com (B.O.); 2 Research Institute for Convergence of Basic Science, Hanyang University, Seoul 04763, Republic of Korea; 3 Research Institute for Natural Sciences, Hanyang University, Seoul 04763, Republic of Korea; 4 Hanyang Institute of Bioscience and Biotechnology, Hanyang University, Seoul 04763, Republic of Korea

**Keywords:** bioluminescence, *Gaussia* luciferase, mutagenesis, coelenterazine, reporter

## Abstract

*Gaussia* luciferase (*G*Luc) is the preeminent secreted luciferase widely used in cell-based reporter assays. By employing sequence-guided mutagenesis informed by alignments of diverse copepod luciferase sequences, we identified key amino acids that significantly enhance bioluminescence (BL) intensity. Among the mutated proteins expressed in bacteria, five individual mutations (M60L, K88Q, F89Y, I90L, or S103T) independently increased BL intensity by 1.8 to 7.5-fold compared to wild-type *G*Luc in the presence of coelenterazine substrates. Remarkably, the combination of all five mutations in *G*Luc (designated as *G*Luc5) resulted in an unexpected 29-fold enhancement in BL intensity. Subsequent evaluation of the *G*Luc5-secreted reporter in transfected mammalian cells confirmed its superior BL performance across multiple cell lines. These findings suggest that the mutated residues are likely crucial for enhancing BL intensity in *G*Luc, supporting its potential to serve as a highly sensitive biosensor or reporter for a wide range of biological applications.

## 1. Introduction

Bioluminescence (BL) assays using luciferases often surpass fluorescence or chemiluminescence assays due to their superior sensitivity, linear dynamic range, and minimal background signal [1,2,3]. Luciferases catalyze the oxidation of substrates (primarily luciferin or coelenterazine) to produce light. To date, luciferases from diverse organisms, including fireflies, *Renilla*, copepods, and bacteria, have been cloned and characterized [4,5,6,7,8]. Firefly luciferase (FLuc), *Renilla* luciferase (*R*Luc), and *Gaussia* luciferase (*G*Luc) are widely employed in bioassays. While FLuc is dependent on adenosine triphosphate (ATP), oxygen, and Mg^2+^ for the oxidation of luciferin, *R*Luc, and *G*Luc can catalyze the oxidation of coelenterazine without requiring ATP or Mg^2+^. Among the coelenterazine-dependent luciferases, *R*Luc has been extensively studied and has a well-characterized structure [9].

*G*Luc, derived from the marine copepod *Gaussia princeps*, has garnered significant attention as a smaller (185 amino acids, ~19.9 kDa) and brighter luciferase compared to FLuc and *R*Luc [8]. The primary advantage of *G*Luc over other luciferases is its natural secretion, enabling non-destructive, real-time monitoring of biological processes in the extracellular medium. This makes *G*Luc particularly valuable for bioanalysis in live cells [10]. Applications of *G*Luc span diverse fields, including live imaging [10,11], protein–protein interactions [12], protein dynamics [13], tumor progression monitoring [14], and high-throughput screening [15]. Along with its advantages, structural and functional understandings of *G*Luc have been reported [16,17,18,19]. Given that *G*Luc’s unique bioluminescence-generating mechanism stems from conformational changes induced by substrate binding rather than multiple catalytic sites [16], we were motivated to engineer novel mutants based on the evolutionary relatedness and sequence similarity of luciferases.

Here, we report on the rational engineering of *G*Luc to enhance BL via sequence-guided mutagenesis. Protein engineering efforts have successfully enhanced and stabilized *R*Luc variants [20,21,22,23]. These modified *R*Luc mutants underpin the development of numerous bimolecular and high-throughput screening assays [24,25]. Similar endeavors have been undertaken with *G*Luc, employing gene shuffling to achieve substantial increases in luminescence [15,26]. Maguire C. A. et al. reported a *G*Luc variant exhibiting glow-type emission kinetics in the presence of the coelenterazine and nonionic surfactant, Triton X-100, suitable for high-throughput screening [15]. Welsh J. P. et al. described a double mutant with an extended luminescence half-life compared to wild-type *G*Luc [27]. Nonetheless, the use of a nonionic surfactant might introduce additional variables and complicate downstream applications, and the overall luminescence intensity of *G*Luc has not been fully explored.

To investigate the functional roles of conserved amino acids in copepod luciferases, we performed a comprehensive sequence analysis, incorporating the known structural data of *G*Luc [17,28]. Through BLAST searches and multiple sequence alignments, we identified highly conserved residues within *G*Luc. These residues were targeted for site-directed mutagenesis, either individually or in combination, and the resulting mutant proteins were expressed in bacteria to assess their impact on BL. The mutant exhibiting the highest BL intensity was further characterized by mammalian cells transfected with the pCMV vector construct.

## 2. Results and Discussion

### 2.1. Comparative Sequence Analysis for Site-Directed GLuc Mutagenesis

**Figure 1:**
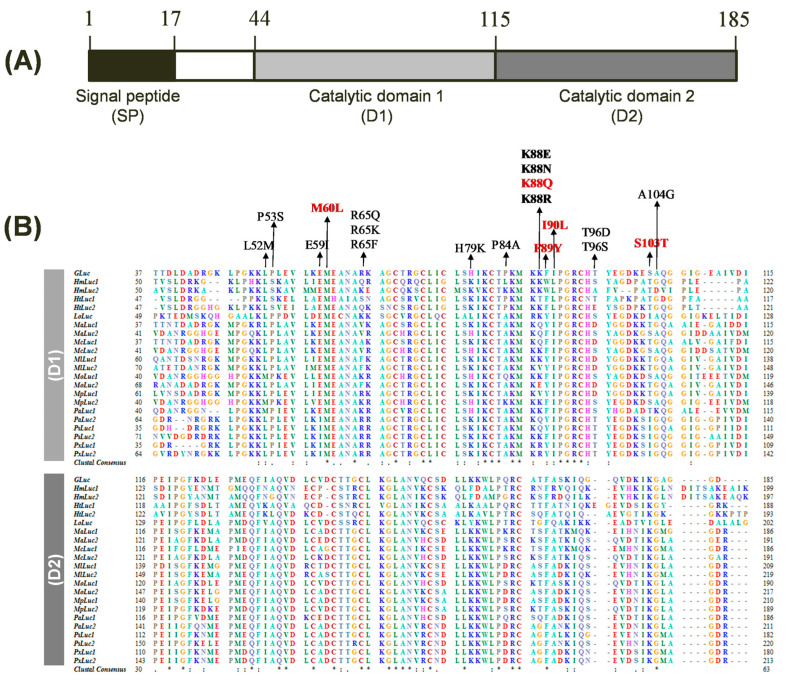
Structure and sequence alignment of *Gaussia* luciferase (*G*Luc). (**A**) Domain architecture of *G*Luc encompassing a signal peptide (SP) and two repeated catalytic domains (D1 and D2). The protein comprises 185 amino acids. (**B**) Multiple sequence alignment of *G*Luc and 21 copepod luciferase homologs, focusing on the D1 (top) and D2 (bottom). Amino acid residues are color-coded based on their physicochemical properties. Identical residues across all sequences are indicated by asterisks (*), while conserved substitutions are represented by a colon (:) and period (.). Gaps introduced for optimal alignment are shown as dashes (–). Amino acid positions are numbered on the left and right. The amino acids targeted for mutagenesis are indicated by arrows, with *G*Luc5 mutation sites highlighted in red.

*G*Luc, a secreted BL reporter protein, has gained widespread recognition due to its exceptional brightness, compact size, and extracellular secretion. However, the precise active site and critical amino acid residues responsible for *G*Luc’s catalytic activity have remained elusive. In 2008, fundamental studies identified two distinct catalytic domains within *G*Luc, where both domains were found to be active when expressed individually [29]. In 2011, a computational analysis suggested that the *G*Luc active site resides within amino acids 71–140, the most hydrophilic region of the protein, based on comparisons with the chromophore region of green fluorescent protein and coelenterazine, along with hydrophobicity analysis [30]. The study also proposed a few mutations (I90L, F89W/I90L, and others) that led to enhanced BL intensities. Recent molecular-directed evolution studies on *G*Luc have identified additional mutations, such as M60I, that result in altered glow-type light emission kinetics [15,26]. Notably, all reported mutations have been confined to the first catalytic domain, and mutating corresponding amino acids in the second domain has not yielded any significant effects [26]. Based on the known structural and functional features of *G*Luc, we conducted a comprehensive sequence analysis of *G*Luc and its homologs. Our analysis identified twenty-one closely related luciferases from the copepod family, sharing approximately 37–73% sequence identity (Table 1). As previously observed [29], the primary structure of *G*Luc exhibits two catalytic domains (D1 and D2) composed of tandem repeat sequences, each consisting of 71 amino acid residues, located at positions 44–114 and 115–185, respectively (Figure 1A). The multiple sequence alignment of *G*Luc with its related copepod luciferases identified several highly conserved residues within these domains, suggesting their potential importance for *G*Luc’s function (Figure 1B). To investigate the functional significance of these consensus residues, we selected several candidate amino acids for mutagenesis based on their sequence conservation and predicted roles in BL activity. Focusing on the first repeated catalytic domain (D1), known to be more essential than D2 [26], we selected 13 amino acid sites (L52, P53, E59, M60, R65, H79, P84, K88, F89, I90, T96, S103, and A104) for mutagenesis within the D1 region (positions 44–114). We included three underlined residues known for their role in enhancing bioluminescence in *G*Luc: M60L [15], F89Y [30], and I90L [30]. The remaining 10 amino acids are either semi-conserved residues from the consensus sequence or frequently substituted residues based on sequence similarity. Cysteine residues in D1 were excluded from mutagenesis due to their structural role rather than catalytic function. Additionally, we excluded amino acids that were identical to the consensus sequence (marked with asterisks) and those previously shown to have no effect on luciferase activity. Mutant *G*Luc proteins with targeted substitutions were expressed, and their BL activity was assessed.

### 2.2. Single Site-Directed Mutagenesis of GLuc for Enhanced BL

**Figure 2:**
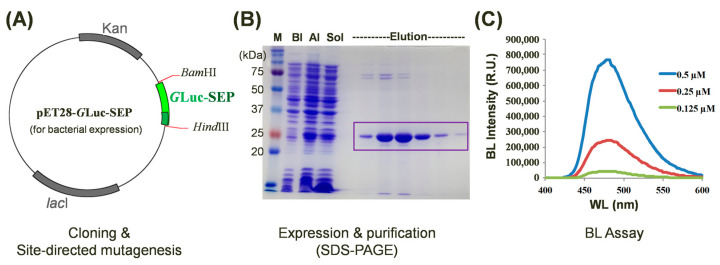
Characterization of site-directed *G*Luc mutants with enhanced bioluminescence (BL) intensity. (**A**) A schematic representation of the pET28-*G*Luc-SEP plasmid used for bacterial expression. The WT or mutated *G*Luc-SEP gene was inserted between *Bam*HI and *Hind*III restriction sites. (**B**) SDS-PAGE analysis of purified *G*Luc-SEP protein. M, BI, AI, and Sol denote size markers before induction, after induction, and as a soluble fraction, respectively. The target protein is indicated by the box. (**C**) Assay of BL intensity of purified *G*Luc-SEP mutants at various concentrations. The BL intensities of the *G*Luc mutants were compared to those of the -type *G*Luc-SEP to verify BL enhancement.

To investigate the impact of single amino acid substitutions on *G*Luc’s BL activity, we generated a series of site-directed mutants derived from the wild-type protein. For the efficient comparison of BL intensities among mutant proteins, we initially constructed a plasmid for bacterial expression. *G*Luc’s expression in bacterial systems is often hindered by the formation of five disulfide bonds because they increase the risk of misfolding when *G*Luc is bacterially produced, resulting in a low yield [31]. For the bacterial expression of *G*Luc, various strategies have been explored to enhance the soluble expression and purification of *G*Luc in bacterial systems [32,33,34]. One effective approach involves the insertion of an SEP-tag (nine Asp residues) at the C-terminus of *G*Luc [17,33]. We incorporated a C-terminal SEP-tag into the pET28 vector (termed pET28-*G*Luc-SEP) and utilized this construct for protein expression and mutagenesis (Figure 2A). The recombinant *G*Luc-SEP protein was successfully expressed in *E. coli* and purified to homogeneity. The molecular weight of the purified *G*Luc protein, as determined by SDS-PAGE analysis, was approximately 24 kDa (Figure 2B), which is consistent with its calculated molecular weight based on the amino acid sequence. To assess the impact of mutations on BL intensity, purified *G*Luc-SEP mutants were subjected to BL assays at varying concentrations (Figure 2C).

**Figure 3:**
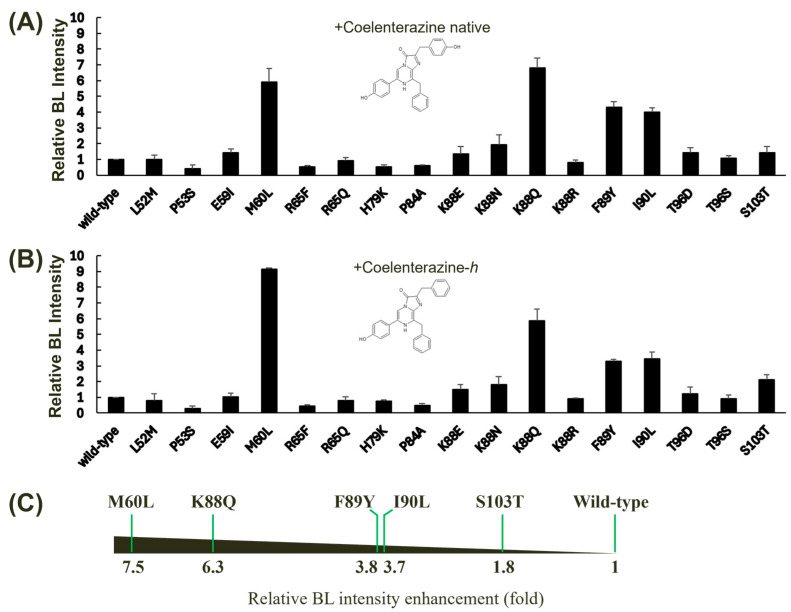
Effect of single mutagenesis on the BL activity of *G*Luc. Single mutants were derived from wild-type *G*Luc via site-directed mutagenesis, and their BL intensities were assessed using two different substrates: coelenterazine native (**A**) and coelenterazine*-h* (**B**). The BL intensity of *G*Luc was measured at 482 nm. Error bars represent the standard deviations from three independent experiments. (**C**) A horizontal bar chart summarizing the relative BL intensity enhancement (fold increase) for selected mutants compared to the wild type is shown. The fold increase was calculated by taking the average of the values in (**A**,**B**). The mutants exhibiting significant increases in BL intensity are displayed.

Despite the high conservation of cysteine residues among copepod luciferases, we excluded them from our site-directed mutagenesis experiments. Previous studies have demonstrated that cysteine residues within *G*Luc play a crucial role in maintaining protein structural stability through disulfide bond formation rather than directly influencing BL activity. It has been reported that the fifth disulfide bond in *G*Luc is dispensable for bioluminescence, as evidenced by complementation experiments involving two inactive *G*Luc domains [35]. The BL intensities of these mutants were measured using two distinct substrates: coelenterazine native (Figure 3A) and coelenterazine-*h* (Figure 3B). Our analysis revealed that several mutations led to significant enhancements in *G*Luc’s BL intensity, particularly when using coelenterazine-*h* as a substrate. This result also suggests that the region encompassing amino acids 60–103 plays a crucial role in substrate specificity, given the structural difference between coelenterazine native and coelenterazine-*h*, which involves an additional −OH group in the native form. Our findings corroborate the hypothesis that the *G*Luc active site resides within amino acids 71–140, as previously suggested, based on hydrophobicity analysis [21]. Collectively, five of the tested single mutants, M60L, K88Q, F89Y, I90L, and S103T, exhibited relatively high increases in BL intensity compared to the wild-type *G*Luc. Among these mutants, M60L and K88Q demonstrated the highest levels of BL enhancement. The M60I substitution has been previously reported to extend the light emission [15]. F89Y and I90L have also been shown to enhance BL in *G*Luc [30]. Notably, the M60L, K88Q, F89Y, and I90L mutants exhibited substantial increases in BL intensity, averaging between 3.7- and 7.5-fold compared to the wild-type protein (Figure 3C). These findings highlight the critical role of these amino acid residues in modulating *G*Luc’s catalytic efficiency and substrate specificity.

### 2.3. Multiple Site-Directed Mutagenesis of GLuc for Enhanced BL

**Figure 4:**
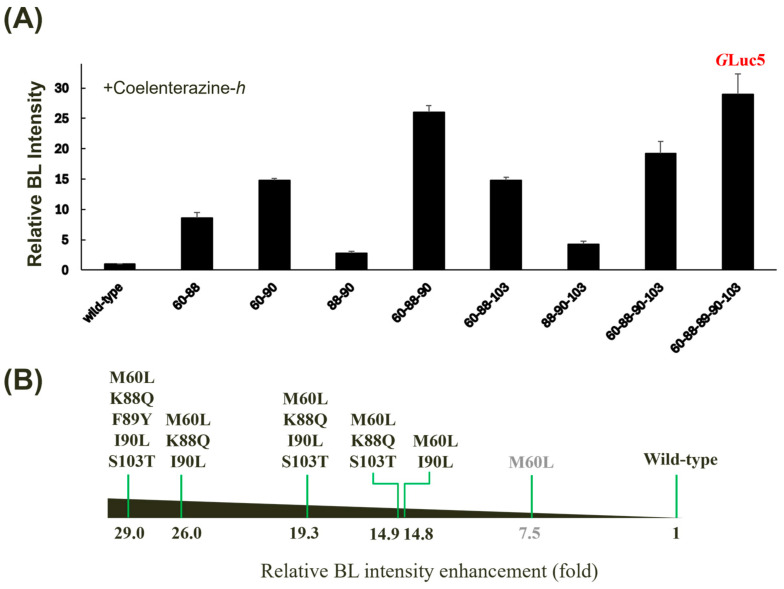
Effect of multiple mutagenesis on the BL activity of *G*Luc. (**A**) Relative BL intensity of multiple mutants compared to the wild type when incubated with coelenterazine-*h*. Error bars represent the standard deviations from three independent experiments. (**B**) A horizontal bar chart summarizing the relative fold increase in BL intensity for selected mutants is shown.

To investigate the synergistic effects of multiple amino acid substitutions on *G*Luc’s BL activity, we generated a series of combinatorial mutants based on the conserved positions (Figure 1B). When evaluated with coelenterazine-*h*, the quintuple mutant (M60L, K88Q, F89Y, I90L, and S103T, denoted *G*Luc5) exhibited the highest BL intensity (~29-fold versus wild-type) among all tested combinations, surpassing every single mutant (Figure 4A). While some double (M60L/I90L), triple (M60L/K88Q/I90L and M60L/L88Q/S103T), or quadruple mutants (M60L/K88Q/I90L/S103T) exhibited significant enhancements in BL activity, some multiple mutant combinations surprisingly exhibited decreased BL intensity compared to their single mutant counterparts (Figure 4B). The M60L substitution appears to be a key determinant of the observed enhancements, as combinations lacking this mutation showed only moderate increases in BL intensity. While a luciferase-based application was not implemented in the current study, *G*Luc5 has the potential to be directly applied in biosensors, as demonstrated by our previous research on measuring protease activity using peptide-linked luciferase [36].

### 2.4. BL Reporter Assay in Cell Culture Medium by Transfection of the GLuc5 Variant

**Figure 5:**
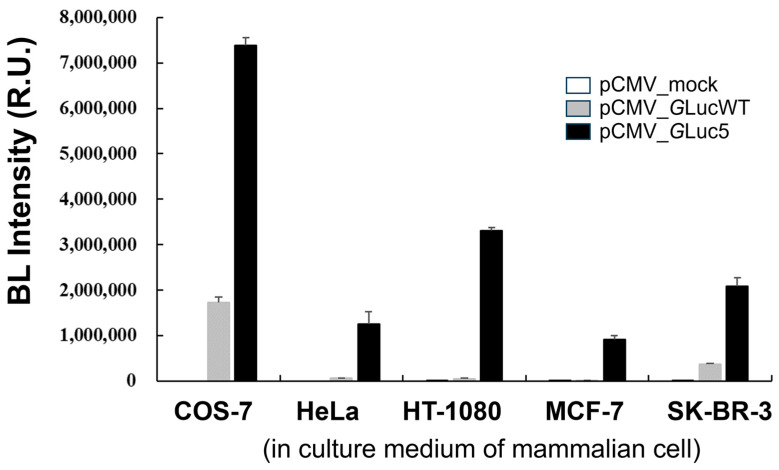
A comparison of BL intensity in mammalian cell lines transfected with pCMV constructs. BL intensity was measured in the culture medium of five mammalian cell lines (COS-7, HeLa, HT-1080, MCF-7, and SK-BR-3) following transfection with an empty pCMV vector (mock, white bar), a pCMV vector expressing wild-type *G*Luc (*G*LucWT, gray bar), or a pCMV vector expressing the *G*Luc5 variant (black bar). Error bars represent the standard deviations from three independent experiments.

The codon-optimized *G*Luc used in the present study has been recognized as a promising reporter protein [8], offering a valuable tool for monitoring various biological processes in conditioned media of cultured cells, as well as in the blood and urine [10,37]. *G*Luc is highly stable in culture media, with a half-life of approximately six days, allowing samples to be stored at 4 °C for several days without a significant loss of reporter activity [37]. To investigate the usefulness of the multi-site-directed mutant *G*Luc5 in mammalian cells, BL intensity was compared across various mammalian cell lines transfected with different pCMV constructs (Figure 5). It is noteworthy that the gene encoding *G*Luc contains a secreted signal peptide sequence at its N-terminus functionally. Among the cell lines tested, COS-7 showed the highest BL intensity, particularly when transfected with the pCMV_*G*Luc5 vector, leading to a substantial increase in BL intensity. Although BL intensity varied widely among different cell lines transfected with the same *G*Luc constructs, other cell lines, including HeLa, HT-1080, MCF-7, and SK-BR-3, also exhibited significant increases in BL intensity when transfected with the pCMV_*G*Luc5 vector compared to the mock and pCMV_*G*LucWT controls. This variability in BL intensity among different cell lines suggests that transfection efficiency, substrate penetration, and/or other cellular factors may influence *G*Luc activity, highlighting the need for further investigation into the underlying mechanisms. Overall, this result indicates that the *G*Luc5 variant significantly enhances BL intensity across different cell lines, supporting its potential as a highly sensitive reporter for a wide range of biological applications, particularly in monitoring gene expression and cellular processes in various cell types. It is noteworthy that compared to the fluorescence measurement, BL offers superior sensitivity and enables the quantification of protein expression at lower concentrations. Consequently, we anticipate that the enhanced sensitivity of the engineered *G*Luc holds significant promise for applications such as BL resonance energy transfer (BRET) and BL imaging.

## 3. Materials and Methods

### 3.1. Materials

The pCMV-*G*Luc plasmid, coelenterazine-native, and coelenterazine-*h* were purchased from Nanolight Technology (Pinetop, AZ, USA) and used for cloning with specific primers. All the primers were sourced from Macrogen (Seoul, Republic of Korea). All other reagents were purchased from commercial suppliers and were of the highest available purity grade.

### 3.2. Sequence-Guided Mutagenesis

A protein-BLAST search was initially conducted using the *G*Luc amino acid sequence to identify related copepod luciferase sequences. Subsequently, multiple sequence alignment was performed with the resulting luciferase sequences from the BLAST search, and highly consensus amino acids were selected for site-directed mutagenesis and characterization. The GenBank accession numbers for the luciferase sequences are as follows: *Metridia asymmetrica* 1 (*Ma*Luc_1, BAN91823), *Metridia asymmetrica* 2 (*Ma*Luc_2, BAN91824), *Metridia curticauda* 1 (*Mc*Luc_1, BAN91825) *Metridia curticauda* 2 (*Mc*Luc_2, BAN91826), *Metridia pacifica* 1 (*Mp*Luc_1, BAG48249), *Metridia pacifica* 2 (*Mp*Luc_2, BAG48250), *Metridia okhotensis* 1 (*Mo*Luc_1, BAM11213), *Metridia okhotensis* 2 (*Mo*Luc_2, BAL63033), *Metridia longa* 1 (*Ml*Luc_1, ABW06650), *M. longa* 2 (*Ml*Luc_2, AAR17541), *Pleuromamma abdominalis* 1 (*Pa*Luc_1, BAL63034), *Pleuromamma abdominalis* 2 (*Pa*Luc_2, BAL63035), *Pleuromamma scutullata* 1 (*Ps*Luc_1, BAN91827), *Pleuromamma scutullata* 2 (*Ps*Luc_2, BAN91828), *P. xiphias* 1 (*Px*Luc_1, BAN91832), *Pleuromamma xiphias* 2 (*Px*Luc_2, BAN91829), *Lucicutia ovaliformis* (*Lo*Luc, BAN91831), *Heterorhabdus tanneri* 1 (*Ht*Luc_1, BAL63039), *Heterorhabdus tanneri* 2 (*Ht*Luc_2, BAL63040), *Heterostylites major* 1 (*Hm*Luc_1, BAL63041), and *Heterostylites major* 2 (*Hm*Luc_2, BAL63042). Site-directed mutagenesis was performed using pET_*G*Luc-SEP plasmid, which carries the *G*Luc gene with a C-terminal solubility enhancement peptide (SEP)-tag. The pET_*G*Luc-SEP was constructed by cloning the *G*Luc sequence from the pCMV-*G*Luc vector. The SEP-tag, consisting of six Asp residues, was added to the C-terminus to enhance solubility, as previously suggested [33], using polymerase chain reaction (PCR). The resulting *G*Luc-SEP was then cloned into the pET28 vector. Single or multiple mutations at pET_*G*Luc-SEP were created by oligonucleotide-directed mutagenesis, as previously described [38]. This method involved the amplification of a target gene with mutagenic primers, followed by the digestion of parental methylated DNA with *Dpn*I. The resulting PCR product, which was not methylated, was then transformed into *E. coli* DH5α cells. Subsequent selection and screening steps allowed for the identification of clones containing the desired mutation. All wild-type and mutant sequences were analyzed using the Macrogen sequencing service in Republic of Korea.

### 3.3. Expression and Purification of GLuc Mutants

The pET28_*G*Luc-SEP expression plasmid was transformed into *E. coli* strain BL21 cells and the transformed bacteria were grown at 37 °C in 250 mL of Luria–Bertani broth containing 50 µg/mL of kanamycin until the optical density at 600 nm reached 0.7. Protein expression was induced by adding 0.5 mM IPTG, and the cells were further incubated for an additional 5 h at 25 °C. Cells were harvested by centrifugation at 7000 rpm for 10 min. The harvested cell pellet was resuspended in a 12.5 mL lysis buffer (50 mM Tris containing 300 mM NaCl, 1 mg/mL lysozyme, 1 mM phenylmethylsulfonyl fluoride, and 0.5% Triton X-100, pH 8.0) and disrupted by ultrasonication. The crude cell extract was centrifuged at 14,000 rpm for 20 min, and the supernatant was filtered and incubated with 1 mL of Ni-NTA beads for 1 h with shaking at 4 °C. The flow-through was removed, and the beads were washed three times with a wash buffer (50 mM Tris containing 300 mM NaCl and 20 mM imidazole, pH 8.0). The bound proteins were eluted with a linear gradient of 0–0.5 M imidazole in a wash buffer. Fractions containing the expressed protein were dialyzed and concentrated in 50 mM Tris (containing 50 mM NaCl, pH 8.0). Mutant proteins were purified in a similar manner. The protein concentration in the soluble fraction was determined using the theoretical extinction coefficient calculated from the amino acid sequences.

### 3.4. Protein-Based BL Assay of Bacteria-Expressed GLuc Mutants

BL assays were conducted in a total volume of 100 µL of 50 mM Tris (containing 50 mM NaCl, pH 8.0) with purified *G*Luc protein and coelenterazine substrates. Briefly, 50 µL of either coelenterazine-native or coelenterazine*-h* solution (final concentration of 10 µg/mL) was mixed with 50 μL of purified *G*Luc (final concentration of 250 nM), and the BL intensity was immediately measured using a plate reader (Varioskan; Thermo Fisher Scientific, Waltham, MA, USA) at a wavelength of 400–600 nm. For analysis, the intensities of the mutant proteins at 482 nm (the wavelength of maximum intensity, λ_max_) were compared with those of the wild type, and enhanced candidates were selected for further characterization.

### 3.5. Secretion-Based BL Assay of Mammalian Cell-Expressed GLuc Mutants

To optimize mammalian cell transfection and luciferase secretion into the culture medium, two plasmids (pCMV_*G*LucWT and pCMV_*G*Luc5) were constructed using the pCMV3_untagged plasmid (Sino Biological, Beijing, China) as a backbone. The DNA sequence encoding *G*Luc_WT (or *G*Luc5 mutant), including the N-terminal signal sequence but excluding the SEP sequence, was amplified from the plasmid pET28_*G*LucWT-SEP (or pET28_*G*Luc5-SEP) by PCR. The forward primer (5′-CCC AAG CTT ATG GGA GTC AAA GTT CTG TTT GCC C-3′) included a *Hind*III restriction site, while the reverse primer (5′-GCT CTA GAT TAG TCA CCA CCG GCC CCC TTG-3′) included a *Xba*I site. Mammalian cell lines (COS-7, HeLa, HT-1080, MCF-7, and SK-BR-3) were cultured in a suitable growth medium with 10% FBS (DMEM for COS-7 and HeLa; RPMI1640 for HT-1080, MCF-7, and SK-BR-7) at 37 °C in a 5% CO_2_ incubator until they reached 70–80% confluence. Cells were transfected with plasmids encoding wild-type *G*Luc (pCMV_*G*LucWT) and the *G*Luc5 mutant (pCMV_*G*Luc5) using the FuGene HD transfection reagent (Promega, Madison, WI, USA) according to the manufacturer’s protocol. The empty plasmid (pCMV_mock) served as a negative control. Cells were transfected for 48 h in a serum-free medium to allow for protein expression. Subsequently, the cell culture supernatant was collected by centrifuging at 300× *g* for 5 min to remove cell debris. The resulting supernatant, containing secreted *G*LucWT or *G*Luc5, was transferred to a clean tube. BL was measured using a microplate reader immediately following the 2:1 mixing of volumes of a culture supernatant (100 µL) and coelenterazine-*h* solution (50 µL) in PBS within a white 96-well plate. The final concentration of coelenterazine-*h* was 6.7 µg/mL. BL intensity was determined at the maximal peak within the 400–600 nm wavelength range.

## 4. Conclusions

Despite its widespread use of *G*Luc in diverse biological applications, its performance can be significantly enhanced through rational protein engineering. We demonstrate that sequence-guided mutagenesis, informed by a comprehensive analysis of copepod luciferases, enabled the development of *G*Luc variants with substantially increased BL intensity. Among the single and multiple mutants generated, individual mutations within the first catalytic domain, including M60L, K88Q, F89Y, I90L, and S103T, were particularly effective at improving BL emissions. Notably, the quintuple mutant *G*Luc5 exhibited a remarkable 29-fold increase in BL intensity compared to the wild-type protein, both in bacterial expression systems and in culture media secreted by five mammalian cell lines. These findings highlight the potential of *G*Luc5 as a promising candidate for a wide range of BL-based applications, including gene expression studies and protein–protein interaction analysis.

## Figures and Tables

**Table 1:** Luciferase sequence similarity among Copepod species revealed by protein BLAST research.

Species Name	Length (a.a.)	Identity (%)	Similarity (%)
*Metridia asymmmetrica*	186	71	84
*Metridia asymmetrica 2*	191	71	83
*Metridia pacifica*	210	68	74
*Metridia pacifica 2*	189	72	82
*Metridia curticauda*	186	71	83
*Metridia curticauda 2*	191	71	82
*Metridia okhotensis*	190	73	82
*Metridia okhotensis 2*	217	63	70
*Metridia longa*	209	64	73
*Metridia longa*	219	58	67
*Pleuromamma scutullata*	182	70	79
*Pleuromamma scutullata 2*	220	61	69
*Pleuromamma abdominalis*	186	70	82
*Pleuromamma abdominalis 2*	211	61	70
*Pleuromamma xiphias*	180	71	80
*Pleuromamma xiphias 2*	213	62	71
*Heterorhabdus tanneri*	188	46	58
*Heterorhabdus tanneri 2*	196	51	65
*Heterostylites major*	205	46	62
*Heterostylites major 2*	203	37	56
*Lucicutia ovaliformis*	223	68	81

## Data Availability

The raw data supporting the conclusions of this article will be made available by the authors on request.

## References

[1] Billard P., DuBow M.S. (1998). Bioluminescence-based assays for detection and characterization of bacteria and chemicals in clinical laboratories. Clin. Biochem..

[2] Kim E.H., Park S., Kim Y.K., Moon M., Park J., Lee K.J., Lee S., Kim Y.P. (2020). Self-luminescent photodynamic therapy using breast cancer targeted proteins. Sci. Adv..

[3] Fan F., Wood K.V. (2007). Bioluminescent assays for high-throughput screening. Assay Drug. Dev. Technol..

[4] Lorenz W.W., McCann R.O., Longiaru M., Cormier M.J. (1991). Isolation and expression of a cDNA encoding *Renilla reniformis* luciferase. Proc. Natl. Acad. Sci. USA.

[5] Markova S.V., Burakova L.P., Vysotski E.S. (2012). High-active truncated luciferase of copepod *Metridia longa*. Biochem. Biophys. Res. Commun..

[6] Takenaka Y., Masuda H., Yamaguchi A., Nishikawa S., Shigeri Y., Yoshida Y., Mizuno H. (2008). Two forms of secreted and thermostable luciferases from the marine copepod crustacean, *Metridia pacifica*. Gene.

[7] Fisher A.J., Thompson T.B., Thoden J.B., Baldwin T.O., Rayment I. (1996). The 1.5-A resolution crystal structure of bacterial luciferase in low salt conditions. J. Biol. Chem..

[8] Tannous B.A., Kim D.E., Fernandez J.L., Weissleder R., Breakefield X.O. (2005). Codon-optimized *Gaussia* luciferase cDNA for mammalian gene expression in culture and in vivo. Mol. Ther..

[9] Loening A.M., Fenn T.D., Gambhir S.S. (2007). Crystal structures of the luciferase and green fluorescent protein from *Renilla reniformis*. J. Mol. Biol..

[10] Tannous B.A. (2009). *Gaussia* luciferase reporter assay for monitoring biological processes in culture and in vivo. Nat. Protoc..

[11] Enjalbert B., Rachini A., Vediyappan G., Pietrella D., Spaccapelo R., Vecchiarelli A., Brown A.J., d’Enfert C. (2009). A multifunctional, synthetic *Gaussia princeps* luciferase reporter for live imaging of *Candida albicans* infections. Infect. Immun..

[12] Remy I., Michnick S.W. (2006). A highly sensitive protein-protein interaction assay based on *Gaussia* luciferase. Nat. Meth..

[13] Kim S.B., Sato M., Tao H. (2009). Split *Gaussia* luciferase-based bioluminescence template for tracing protein dynamics in living cells. Anal. Chem..

[14] Chung E., Yamashita H., Au P., Tannous B.A., Fukumura D., Jain R.K. (2009). Secreted *Gaussia* luciferase as a biomarker for monitoring tumor progression and treatment response of systemic metastases. PLoS ONE.

[15] Maguire C.A., Deliolanis N.C., Pike L., Niers J.M., Tjon-Kon-Fat L.A., Sena-Esteves M., Tannous B.A. (2009). *Gaussia* luciferase variant for high-throughput functional screening applications. Anal. Chem..

[16] Larionova M.D., Markova S.V., Vysotski E.S. (2018). Bioluminescent and structural features of native folded *Gaussia* luciferase. J. Photoch. Photobio. B.

[17] Wu N., Kobayashi N., Tsuda K., Unzai S., Saotome T., Kuroda Y., Yamazaki T. (2020). Solution structure of Luciferase with five disulfide bonds and identification of a putative coelenterazine binding cavity by heteronuclear NMR. Sci. Rep..

[18] Dijkema F.M., Nordentoft M.K., Didriksen A.K., Corneliussen A.S., Willemoës M., Winther J.R. (2021). Flash properties of *Gaussia* luciferase are the result of covalent inhibition after a limited number of cycles. Protein Sci..

[19] Ohmuro-Matsuyama Y., Matsui H., Kanai M., Furuta T. (2023). Glow-type conversion and characterization of a minimal luciferase via mutational analyses. FEBS J..

[20] Loening A.M., Dragulescu-Andrasi A., Gambhir S.S. (2010). A red-shifted Renilla luciferase for transient reporter-gene expression. Nat. Meth..

[21] Loening A.M., Fenn T.D., Wu A.M., Gambhir S.S. (2006). Consensus guided mutagenesis of *Renilla* luciferase yields enhanced stability and light output. Protein Eng. Des. Sel..

[22] Loening A.M., Wu A.M., Gambhir S.S. (2007). Red-shifted *Renilla reniformis* luciferase variants for imaging in living subjects. Nat. Meth..

[23] Woo J., von Arnim A.G. (2008). Mutational optimization of the coelenterazine-dependent luciferase from *Renilla*. Plant Meth..

[24] Dragulescu-Andrasi A., Chan C.T., De A., Massoud T.F., Gambhir S.S. (2011). Bioluminescence resonance energy transfer (BRET) imaging of protein-protein interactions within deep tissues of living subjects. Proc. Natl. Acad. Sci. USA.

[25] Bacart J., Corbel C., Jockers R., Bach S., Couturier C. (2008). The BRET technology and its application to screening assays. Biotechnol. J..

[26] Degeling M.H., Bovenberg M.S., Lewandrowski G.K., de Gooijer M.C., Vleggeert-Lankamp C.L., Tannous M., Maguire C.A., Tannous B.A. (2013). Directed molecular evolution reveals *Gaussia* luciferase variants with enhanced light output stability. Anal. Chem..

[27] Welsh J.P., Patel K.G., Manthiram K., Swartz J.R. (2009). Multiply mutated *Gaussia* luciferases provide prolonged and intense bioluminescence. Biochem. Biophys. Res. Commun..

[28] Dijkema F.M., Escarpizo-Lorenzana M.I., Nordentoft M.K., Rabe H.C., Sahin C., Landreh M., Branca R.M., Sorensen E.S., Christensen B., Prestel A. (2024). A suicidal and extensively disordered luciferase with a bright luminescence. Protein Sci..

[29] Inouye S., Sahara Y. (2008). Identification of two catalytic domains in a luciferase secreted by the copepod Gaussia princeps. Biochem. Biophys. Res. Commun..

[30] Kim S.B., Suzuki H., Sato M., Tao H. (2011). Superluminescent variants of marine luciferases for bioassays. Anal. Chem..

[31] Baneyx F., Mujacic M. (2004). Recombinant protein folding and misfolding in *Escherichia coli*. Nat. Biotechnol..

[32] Goerke A.R., Loening A.M., Gambhir S.S., Swartz J.R. (2008). Cell-free metabolic engineering promotes high-level production of bioactive *Gaussia princeps* luciferase. Metab. Eng..

[33] Rathnayaka T., Tawa M., Nakamura T., Sohya S., Kuwajima K., Yohda M., Kuroda Y. (2011). Solubilization and folding of a fully active recombinant *Gaussia* luciferase with native disulfide bonds by using a SEP-Tag. Biochim. Biophys. Acta..

[34] Rathnayaka T., Tawa M., Sohya S., Yohda M., Kuroda Y. (2010). Biophysical characterization of highly active recombinant *Gaussia* luciferase expressed in *Escherichia coli*. Biochim. Biophys. Acta..

[35] Markova S.V., Larionova M.D., Vysotski E.S. (2019). Shining light on the secreted luciferases of marine copepods: Current knowledge and applications. Photochem. Photobiol..

[36] Nguyen D.L., Kim H., Kim D., Lee J.O., Gye M.C., Kim Y.P. (2018). Detection of matrix metalloproteinase activity by bioluminescence via intein-mediated biotinylation of luciferase. Sensors.

[37] Wurdinger T., Badr C., Pike L., de Kleine R., Weissleder R., Breakefield X.O., Tannous B.A. (2008). A secreted luciferase for monitoring of processes. Nat. Meth..

[38] Weiner M.P., Costa G.L., Schoettlin W., Cline J., Mathur E., Bauer J.C. (1994). Site-directed mutagenesis of double-stranded DNA by the polymerase chain-reaction. Gene.

